# Redescription of two Pennellids (Copepoda, Siphonostomatoida) from Korea with a key to species of *Peniculus* von Nordmann, 1832

**DOI:** 10.3897/zookeys.243.3668

**Published:** 2012-11-16

**Authors:** B. A. Venmathi Maran, Seong Yong Moon, Sung-Yong Oh, Ho Young Soh, Jung-Goo Myoung

**Affiliations:** 1 Marine Ecosystem Research Division, Korea Institute of Ocean Science & Technology, P. O. Box 29, Seoul 425-600, Republic of Korea; 2 Faculty of Marine Technology, Chonnam National University, Yeosu, Jeollanamdo 550-749, Republic of Korea

**Keywords:** Copepod, pennellid, parasite, redescription, black scraper, rockfish, fins, identification, key

## Abstract

Redescriptions of two pennellid copepods, *Peniculus minuticaudae* Shiino, 1956 and *Peniculus truncatus* Shiino, 1956, are provided, based on postmetamorphic adult females collected from marine ranched fishes captured at Tongyeong marine living resources research & conservation center, Korea. *Peniculus minuticaudae* was collected from the soft fin rays of black scraper *Thamnaconus modestus*. It can be distinguished from the other two closely related congeners *Peniculus ostraciontis* Yamaguti, 1939 and *Peniculus truncatus* by having a well developed triangular-shaped abdomen; the abdomen is rudimentary in other two species. This is thefirst report of the occurrence of *Peniculus minuticaudae* in Korea. *Peniculus truncatus* was collected from the dorsal fin of Korean rockfish *Sebastes schlegelii*. It can be distinguished from *Peniculus minuticaudae* by the combination of a rudimentary abdomen, long neck and setae on leg 1 and from *Peniculus ostraciontis* by the long neck, slender trunk, and setae on leg 1. It is also shown that *Peniculus truncatus* captured from the same host in Korea was misidentified as *Peniculus ostraciontis* and hence, this is thesecond record of the occurrence of *Peniculus truncatus* in Korea. A key is provided for the 14 nominal species of *Peniculus*.

## Introduction

The genus *Peniculus* von Nordmann, 1832 belongs to the family Pennellidae Burmeister, 1835 and contains 14 nominal species (Boxshall and Halsey 2004). Pennellids are highly transformed, often elongated copepods parasitic on marine fishes and cetaceans (Kabata 1979). Some of pennellids are ectoparasitic (e.g. *Exopenna* Boxshall, 1986; *Parinia* Kazachenko & Avdeev, 1977) but many are deeply inserted into the body of their host. The insertion can take place in the gills, the skin or in the musculature of the host without any particular preference, as is the case for the genus *Pennella* Oken, 1816 (Kabata 1981; Boxshall 1986).

Two species of *Peniculus* are redescribed from Korea in this study. They are *Peniculus minuticaudae* Shiino, 1956 and *Peniculus truncatus* Shiino, 1956. In Asia, nine species of *Peniculus* have so far been reported including six from India and three from Japan. The species reported from Japan are *Peniculus minuticaudae*, *Peniculus truncatus* and *Peniculus ostraciontis* Yamaguti, 1939 (Shiino 1956, 1959; Yamaguti 1939, 1963). One of these three pennellids, *Peniculus ostraciontis*, was redescribed from Korea by Choi et al. (1996) but we reveal here that theirs was a misidentification of *Peniculus truncatus*.

Shiino (1956) described *Peniculus minuticaudae* based on females collected from the fins of threadsail filefish *Stephanolepis cirrhifer* (Temminck and Schlegel, 1850) (*= Monacanthus cirrhifer*), from Shirahama, Wakayama Prefecture, Japan. Recently, infection of *Peniculus minuticaudae* on two cultured fish hosts, *Stephanolepis cirrhifer* and the black scraper *Thamnaconus modestus* (Günther, 1877), was reported from Oita Prefecture, Japan (Nagasawa et al. 2011), after Fukuda (1999) reported the same species from the same locality as an unidentified *Peniculus* sp.

*Peniculus truncatus* was also identified and described by Shiino (1956) based on a single female found on the fin ray of oblong rockfish *Sebastes oblongus* Günther (1877) [= *Sebastichthys mitsukurii*] collected off Wagu, Mie Prefecture, Japan. A third species, *Peniculus ostraciontis*,was described based on females collected from the head of Humpback turretfish *Tetrosomus gibbosus* (Linnaeus, 1758) [= *Ostracion gibbosum*] on the Pacific coast of Japan (Yamaguti 1939). It was reported again from the triangular boxfish *Tetrosomus concatenatus* (Bloch, 1785) [= *Rhinesomus concatenatus*] from Sagami Bay by Shiino (1959) (Table 1). All three *Peniculus* species are in need of redescription and here we undertake the redescription of two of them.

The host *Tetrosomus modestus* have been cultured at a few localities along the southern coastal regions of Korea. At Tongyeong marine living resources research & conservation center (TMRC), several commercially important fishes were ranched under the marine ranching program in Korea by Korea Institute of Ocean Science & Technology (KIOST) from 1998 (MOMAF 2007). Recently, we studied the symbiotic organisms associated with ranched fishes and their life cycles at TMRC (Venmathi Maran et al. 2012). The black scraper is one of the fishes that have been transferred into cages for the purpose of experimentally studying its feeding activities within this marine ranching program. The second host, *Stephanolepis cirrhifer*, is uncommon in culture in Korea because of its small size and low growth rate, in contrast to Japan (Fukuda 1999). The Korean rockfish *Sebastes schlegelii* Hilgendorf, 1880 has been cultured at several localities around the southern coastal region of Korea due to its high commercial value (MOMAF 2007). Despite the increasing threat of parasites in aquaculture, information on parasites and diseases are largely lacking from farmed fishes in Korea. The redescription of *Peniculus minuticaudae* and *Peniculus truncatus* is necessary to reveal previously omitted or overlooked features of both species and also to correct the misidentification by Choi et al. (1996) in Korea. In addition, a key is provided for all 14 nominal species of *Peniculus*.

**Table 1.** Hosts and localities of collections of Pennellids (Copepoda: Siphonostomatoida) from Korea and Japan.

**Pennellid**	**Host**	**Infected site**	**Host order: family**	**Locality**	**Reference**
*Peniculus minuticaudae* Shiino, 1956	*Stephanolepis cirrhifer* (Temminck and Schlegel, 1850) [= *Monacanthus cirrhifer*]	Fins	Tetraodontiformes: Monocanthidae	Shirahama, Wakayama Prefecture, Japan	Shiino 1956
	*Stephanolepis cirrhifer*	Fins	Monocanthidae	Oita Prefecture, Japan	Nagasawa et al. 2011
	*Thamnaconus modestus* (Günther, 1877)	Fins	Monocanthidae	Oita Prefecture, Japan	Nagasawa et al. 2011
	*Thamnaconus modestus*	Fins	Monocanthidae	Tongyeong, Gyeongsangnam-do, Korea	Present study
*Peniculus ostraciontis* Yamaguti, 1939	*Tetrosomus gibbosus* (Linnaeus, 1758) [= *Ostracion gibbosum*]	Head	Tetraodontiformes: Ostraciidae	Pacific Ocean, Aziro, Kanagawa Prefecture, Japan	Yamaguti 1939
	*Tetrosomus concatenatus* (Bloch, 1785) [= *Rhinesomus concatenatus*]	Head	Ostraciidae	Sagami Bay, Japan	Shiino 1959
*Peniculus truncatus* Shiino, 1956	*Sebastes oblongus* (Günther, 1877) [= *Sebastichthys mitsukurii*]	Fins	Scorpaeniformes: Sebastidae	Off Wagu, Mie Prefecture, Japan	Shiino 1956
	*Sebastes schlegelii* Hilgendorf, 1880	Fins	Sebastidae	Haklim fish farm, Kamak Bay, Jeollanam-do, Korea	Choi et al. 1996
	*Sebastes schlegelii*	Dorsal Fin	Sebastidae	Tongyeong, Gyeongsangnam-do, Korea	Present study

## Materials and methods

**Figure 1.**
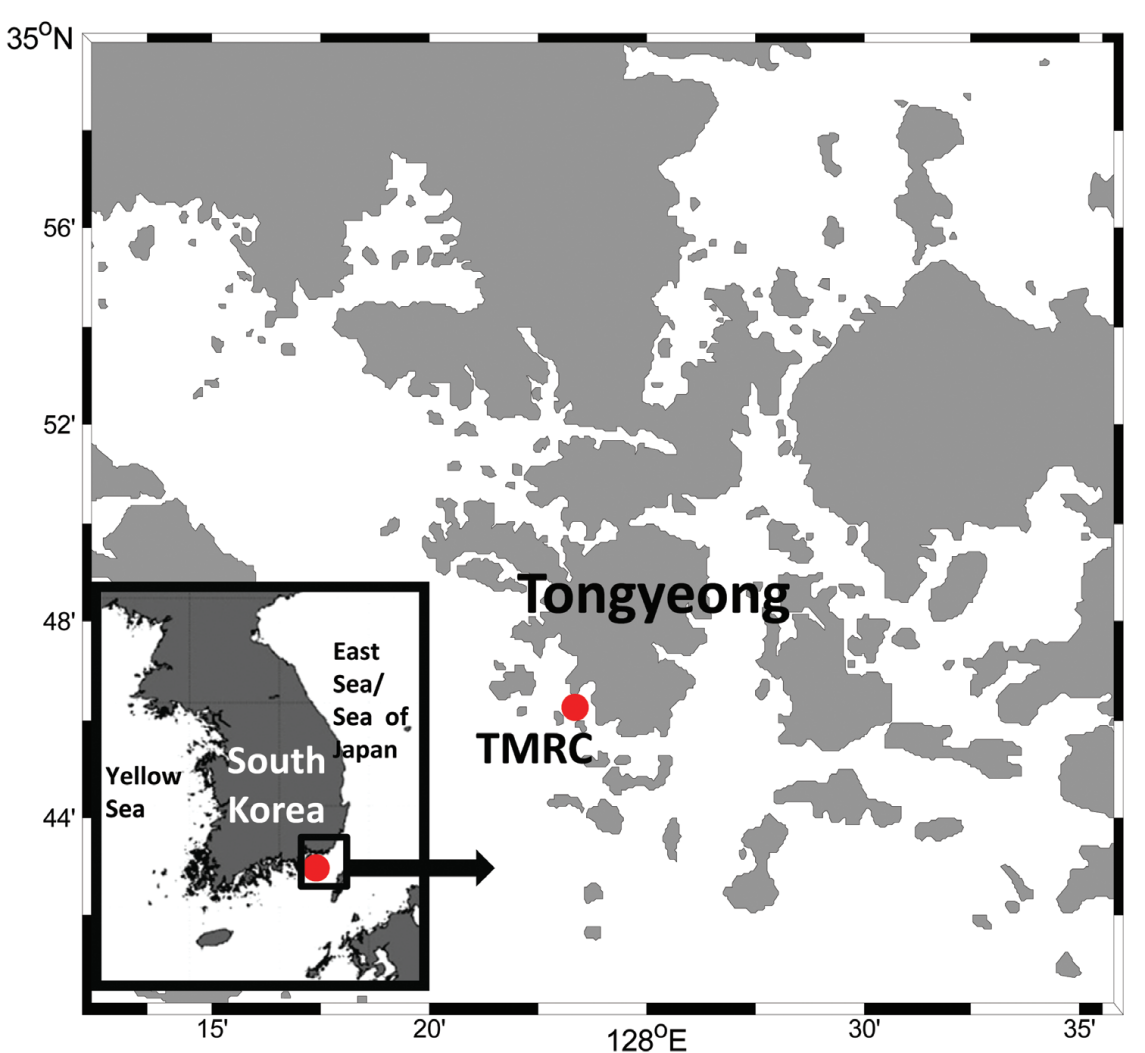
Map showing the marine ranched fish farming facility, Tongyeong marine living resources research & conservation center (TMRC), Tongyeong, Gyeongsangnam-do, Korea

The pennellids were carefully removed from the fin rays of the marine ranched *Tetrosomus modestus* and *Stephanolepis schlegelii* at TMRC, Tongyeong, Gyeongsangnam-do, Korea (Figure 1) and they were preserved in 70% ethanol. Preserved copepods were cleared in a drop of 85% lactic acid or lactophenol prior to examination using an Olympus BX51 phase contrast microscope. Selected specimens were measured intact using an ocular micrometer and/or dissected and examined according to the wooden slide procedure of Humes and Gooding (1964). Measurements given are the mean followed by the range in parentheses. Drawings were made with the aid of a drawing tube. The descriptive terminology follows Kabata (1979) and the common and scientific names of host fishes follow FishBase (Froese and Pauly 2012). Voucher specimens are deposited at the National Institute of Biological Resources (NIBR), Incheon and Marine Biodiversity Institute of Korea (MABIK), Seocheon, Korea.

## Results

### Order Siphonostomatoida Burmeister, 1835

Family Pennellidae Burmeister, 1835

Genus *Peniculus* von Nordmann, 1832

#### Peniculus minuticaudae

Shiino, 1956

http://species-id.net/wiki/Peniculus_minuticaudae

Figures 2 3

Peniculus minuticaudae Shiino, 1956: 593; Nagasawa et al. 2011: 43; Yamaguti 1963: 1104.
Peniculus sp. Fukuda 1999: 57.

##### Material examined.

10 ♀♀ (NIBRIV0000245080) and 2 ♀♀ (MABIK CR00178439) from *Thamnaconus modestus*, Tongyeong, Gyeongsangnam-do, Korea, 20 September 2011.

##### Description.

**Figure 2.**
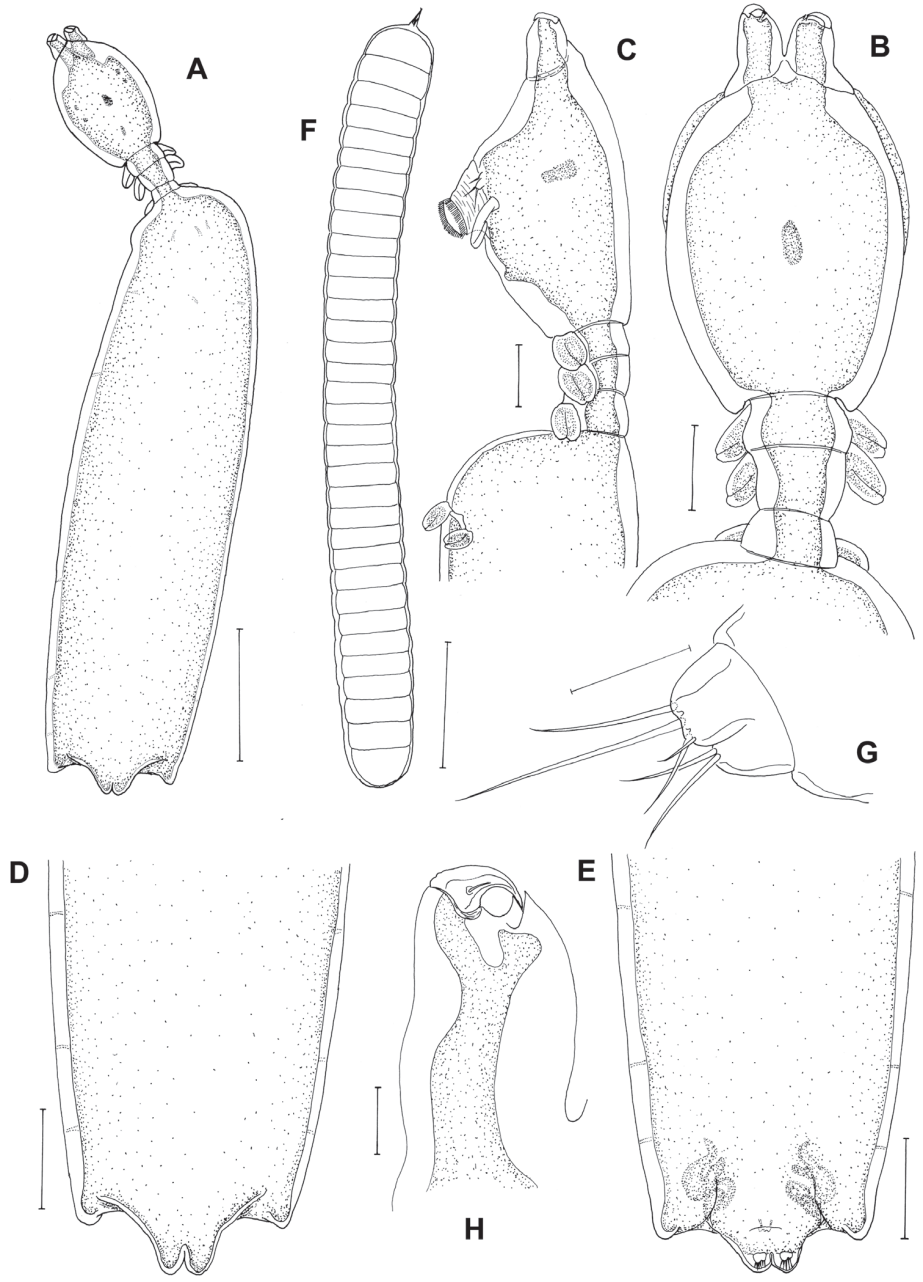
*Peniculus minuticaudae* Shiino, 1956. Postmetamorphic adult female. **A** Habitus, dorsal **B** Cephalothorax and free thoracic somites, dorsal **C** Cephalothorax and free thoracic somites, lateral **D** Posterior end of trunk with abdomen, dorsal **E** Posterior end of trunk with abdomen, ventral **F** Egg sac **G** Caudal ramus **H** Antenna, dorsal. Scale bars: **A**=500 μm; **B–F**=200 μm; **G**=25 μm; **H**=50 μm.

*Postmetamorphic adult female*. Body (Figure 2A), 2.42 (2.12–2.73) mm long (n=10) comprising oval head, slender neck, large trunk and reduced abdomen. Head (cephalothorax) ovoid, longer than wide, with blunt pointed apex (Figure 2B, C). Short slender neck (Figure 2C) consisting of three somites bearing legs 1, 2 and 3. Fourth pedigerous somite incorporated into trunk. Trunk large, cylindrical, longer than wide, bearing leg 4 proximally (Figure 2C). Abdomen slightly triangular-shaped (Figure 2D, E) long with subterminal caudal rami on ventral surface and projecting posterior tip with anal indentation. Egg sacs long and uniseriate with 33–40 eggs (Figure 2F). Caudal rami (Figure 2G) bearing 2 long, 3 medium sized subequal, 1 small setae. Antennule not observed. Antenna (Figure 2H) 2-segmented, chelate; proximal segment consisting of 2 pointed projections overlapping each other; terminal segment claw-like, acutely pointed with minute seta at base.

**Figure 3.**
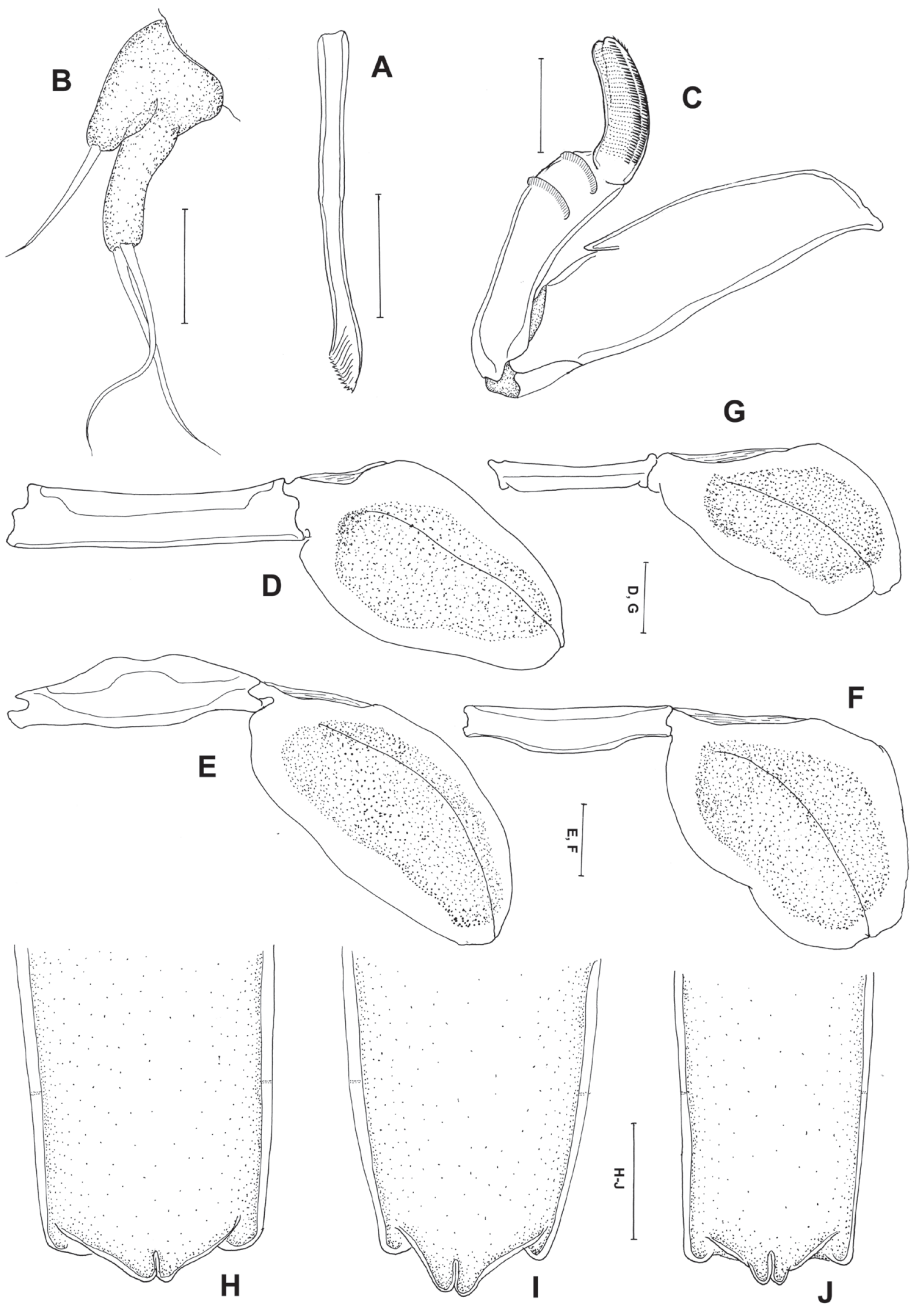
*Peniculus minuticaudae* Shiino, 1956. Postmetamorphic adult female. **A** Mandible, ventral **B** Maxillule, ventral **C** Maxilla, dorsal **D** Leg 1, ventral **E** Leg 2, ventral **F** Leg 3, ventral **G** Leg 4, ventral **H–J** variations of posterior end of trunk with abdomen, dorsal. Scale bars: **A–C**=25 μm; **D–G**=50 μm; **H–J**=200 μm.

Mandible (Figure 3A) broad with 10 teeth terminally. Maxillule (Figure 3B) with 2 lobes having one and two long setae. Maxilla (Figure 3C) 2-segmented; proximal segment broad with spiniform small process, 2 rows of setules distally; distal segment blunt and curved with transverse striations and rows of spinules. Maxilliped absent. Legs 1 to 4 (Figure 3D–G) all represented by broad plate-like structures derived from the protopodal segments, without rami or seta. Leg 5 absent.

##### Variability.

Some females showed variation on posterior end of trunk and abdomen (Figure 3H–J).

##### Attachment site.

All fins of host fish.

##### Remarks.

Careful comparison between our material and the original description of *Peniculus minuticaudae* provided by Shiino (1956) revealed some differences: (1) the abdomen was described as trapezoid and rhomboid; (2) the striation and fine setulose ornamentation of the maxilla was not shown. The mandible was not described. Our redescription revealed that the abdomen of *Peniculus minuticaudae* is triangular and protrudes, however, the two closely related congeners *Peniculus ostraciontis* and *Peniculus truncatus* both have a rudimentary abdomen. We also noted some variation in the posterior end of trunk and abdomen (Figure 3H–J). In the maxilla, fine striations and rows of setulose were found on the distal segment. In addition, the trunk is long and narrow in *Peniculus minuticaudae* and there is no major gap between cephalothorax and trunk so it has a short neck, where legs 1 to 3 are located (Figure 2C). Leg 4 (Figure 2C) is embedded on the anterior part of the trunk. In comparison, the closely related congener *Peniculus ostraciontis* has a stout trunk and short neck (Yamaguti 1939) while *Peniculus truncatus* has a long trunk and neck, and leg 1 has minute setal structure which are not present in *Peniculus minuticaudae* and *Peniculus ostraciontis*.

#### Peniculus truncatus

Shiino, 1956

http://species-id.net/wiki/Peniculus_truncatus

Figures 4 5

Peniculus truncatus Shiino, 1956: 593; Yamaguti 1963: 1104.
Peniculus ostraciontis : Choi et al. 1996: 117.

##### Material examined.

4 ♀♀ (NIBRIV0000252624) and 1 ♀ (MABIK CR00178440) from *Sebastes schlegelii*, Tongyeong, Gyeongsangnam-do, Korea, 15 February 2012.

##### Description.

**Figure 4.**
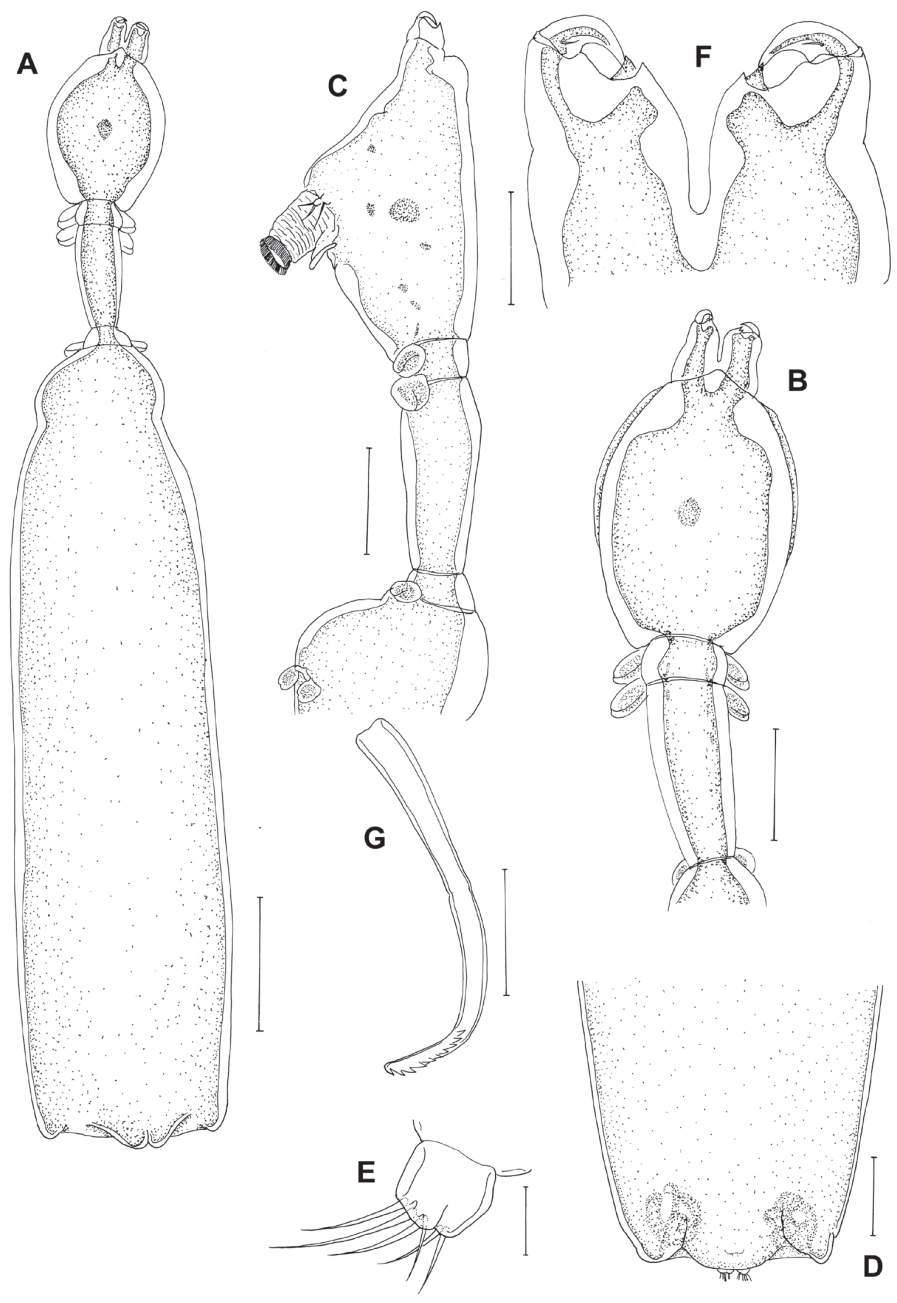
*Peniculus truncatus* Shiino, 1956. Postmetamorphic adult female. **A**. Habitus, dorsal **B** Cephalothorax and free thoracic somites, lateral **C** Cephalothorax and free thoracic somites, dorsal **D** Posterior end of trunk with abdomen, ventral **E** Caudal ramus, ventral **F** Antenna, dorsal **G** Mandible, ventral. Scale bars: **A**=500, μm; **B–D**=200 μm; **E, G**=25 μm; **F**=50 μm.

*Postmetamorphic adult female*. Body (Figure 4A), 4.59 (4.14–5.41) mm long (n=4) comprising oval head, long slender neck, large trunk and reduced abdomen. Head (cephalothorax) ovoid, flattened dorsally but convex ventrally with pair of rounded swellings anteriorly bearing antennae (Figure 4B, C). Mouth tube prominent, directed posteroventrally (Figure 4C). Neck long (0.47–0.55 mm) (Figure 4B, C), slender, comprising about one sixth of trunk length; consisting of three somites bearing legs 1, 2 and 3 (Figure 4B, C). Fourth pedigerous somite incorporated into trunk. Trunk slender, cylindrical, longer than wide, 6 times longer than neck, bearing leg 4 proximally. Abdomen (Figure 4D), reduced with subterminal caudal rami on ventral surface. Caudal rami (Figure 4E) bearing 6 setae. Egg sacs long and uniseriate with 30-37 eggs. Antennule not observed. Antenna (Figure 4F) 2-segmented, chelate; proximal segment bearing 2 pointed projections overlapping each other; terminal segment claw-like, acutely pointed with minute seta at base. Mandible (Figure 4G) moderate-sized, broad, provided with 10 teeth terminally.

**Figure 5.**
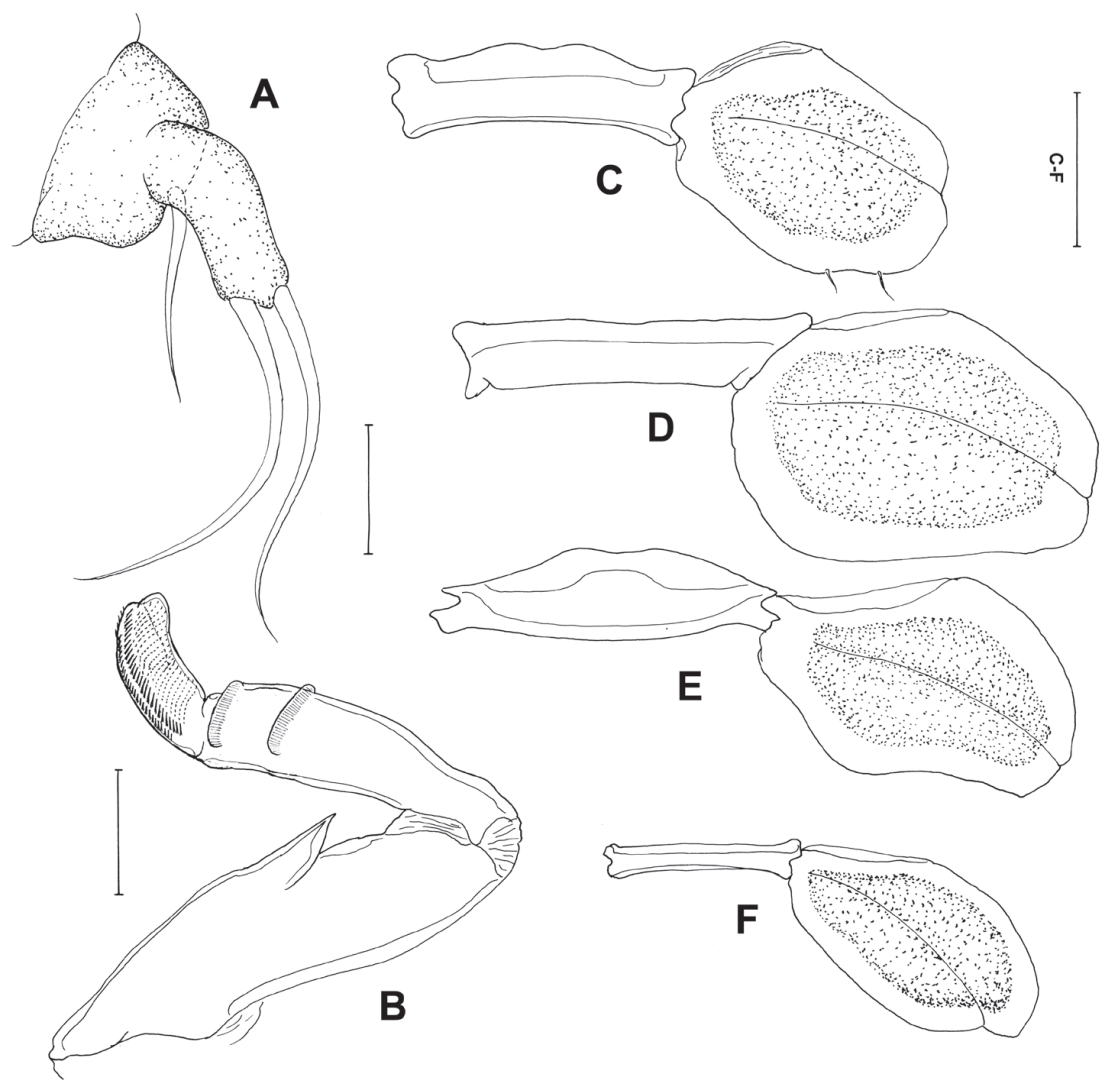
*Peniculus truncatus* Shiino, 1956. Postmetamorphic adult female. **A** Maxillule, dorsal **B** Maxilla, dorsal **C** Leg 1, ventral **D** Leg 2, ventral **E** Leg 3, ventral **F** Leg 4, ventral. Scale bars: **A, B**=25 μm; **C–F**=50 μm.

Maxillule (Figure 5A) with 2 lobes having one short and two long setae. Maxilla (Figure 5B) 2-segmented; proximal segment broad with robust spiniform process, projecting laterally, 2 rows of setules distally; distal segment blunt and curved with transverse striations and rows of spinules. Maxilliped absent. Leg 1 (Figure 5C) forming blunt plate-like structure derived from protopodal segments, with 2 minute setae laterally. Legs 2–4 (Figure 5D–F) as for leg 1, but without seta. Leg 5 absent.

##### Attachment site.

Only on dorsal fin-rays.

##### Remarks.

Comparison between our material and the original description of *Peniculus truncatus* provided by Shiino (1956) revealed some omissions in that the antennae and mandibles were not shown, and possible differences, since the striation of setules on maxilla was not shown. The characteristic features of *Peniculus truncatus* are: (1) the rudimentary abdomen; (2) the long neck (more than half as long as cephalothorax); (3) the maxilla with transverse striations of setules and rows of spinules on the distal segment; (4) the leg 1 is tipped with 2 minute setae laterally. *Peniculus truncatus* differs from *Peniculus minuticaudae* in its rudimentary abdomen (vs. well developed abdomen); long neck (vs. short neck); and in the presence of setae on leg 1 (vs. absence of seta). It differs from *Peniculus ostraciontis* in its moderately slender trunk (vs. stout trunk); long neck, ie: neck more than half as long as cephalothorax (vs. short neck, ie: neck less than half as long as cephalothorax); and in the presence of setae on leg 1 (vs. absence of setae) (Yamaguti 1939; Shiino 1956).

Choi et al. (1996) reported the same pennellid collected from the fins of *Stephanolepis schlegelii* as *Peniculus ostraciontis*. We compared our material with their illustrations (specimens were not deposited in the museum). It showed the features of *Peniculus truncatus*: (1) long neck; (2) slender trunk [not as stout as like *Peniculus ostraciontis* illustrated by Yamaguti (1939)] and the host was *Stephanolepis schlegelii* (Choi et al. 1996), as in the present study.

## Discussion

According to Boxshall and Halsey (2004), there are 14 species on the genus *Peniculus*: *Peniculus asinus* Kabata & Wilkes, 1977; *Peniculus clavatus* (Müller, 1779); *Peniculus communis* Leigh-Sharpe, 1934; *Peniculus elegans* Leigh-Sharpe, 1934; *Peniculus elongatus* Boxshall, 1986; *Peniculus fistula* Nordmann, 1832; *Peniculus furcatus* Krøyer, 1863; *Peniculus minuticaudae* Shiino, 1956; *Peniculus ostraciontis* Yamaguti, 1939; *Peniculus scomberi* Gnanamuthu, 1951; *Peniculus stromatei* Gnanamuthu, 1951; *Peniculus theraponi* Gnanamuthu, 1951; *Peniculus trichuri* Gnanamuthu, 1951; and *Peniculus truncatus* Shiino, 1956.Seven of these are reported from Asian countries.

Alexander (1983) reported *Peniculus haemuloni* from Brazil, however, it was subsequently treated as a separate genus, *Metapeniculus* Castro-Romero & Baeza-Kuroki, 1985 based on the presence of only 3 pairs of swimming legs (vs. 4 pairs for *Peniculus*) (Boxshall, 1986). According to Kabata (1979), two species of *Peniculus*, *Peniculus calamus* Nordmann, 1864 and *Peniculus fissipes* Wilson, 1917 should be regarded as *species inquirendae*, and in addition we treat *Peniculus sciaenae* Gnanamuthu, 1951 as *species inquirenda* since it is also reported with 3 pairs of swimming legs (Gnanamuthu 1951a; Alexander 1983). Thus there are 14 species considered valid and a key is provided for nominal species following Alexander (1983). Most *Peniculus* species were not described adequately by modern standards and most need to be redescribed. In Asia, all species are poorly described and detailed studies are necessary for the five species reported from India (Gnanamuthu 1951a; 1951b; Pillai 1985) and for the three from Japan (Yamaguti 1939; Shiino 1956).

The mean body length of *Peniculus minuticaudae* was 2.42 mm. It corresponds well to the body length (2.48 mm) of *Peniculus minuticaudae* reported from Oita Prefecture, Japan (Nagasawa et al. 2011). The morphological features (Figures 2, 3) agree with the original description of *Peniculus minuticaudae* (Shiino 1956). The present collection represents the first record of *Peniculus minuticaudae* from ranched *Tetrosomus modestus* in Korea. Thus, it is the third documented record of pennellid copepod from commercially cultured fishes.

*Peniculus truncatus* was originally reported from *Stephanolepis oblongus* in Japan (Shiino 1956). This parasite is shown here to utilize a second host species, *Stephanolepis schlegelii*, of the same host genus, although it was initially misidentified as *Peniculus ostraciontis* by Choi et al. (1996). The misidentification was revealed by comparison between Choi’s descriptions, our material and Yamaguti (1939) illustrations of *Peniculus ostraciontis*. We collected *Peniculus truncatus* from the same host species *Stephanolepis schlegelii* cultured in Korea. The host for *Peniculus ostraciontis* is *Tetrosomus gibbosus* (Table 1). In Choi et al. (1996) redescription, they overlooked the third seta on the maxillule and the setules on the maxilla, in addition to the minute setal structures on leg 1.

*Peniculus truncatus* has so far been reported from two species of the genus *Sebastes*, *Stephanolepis schlegelii and S. oblongus* and this pennellid appears to be host specific to rockfish (Table 1). *Peniculus minuticaudae* and *Peniculus ostraciontis* might be specific to file fish and puffer hosts, respectively (Yamaguti 1939; Shiino 1956; 1959; Nagasawa et al. 2011; present study). A key is provided for all 14 valid species below.

### Key to the species of *Peniculus*

(Modified from Alexander 1983)

**Table:** 

1	Cephalothorax with 4 large holdfast processes	*Peniculus asinus* Kabata
–	Cephalothorax without such processes	2
2	Cephalothorax with rounded swelling on ventral surface posterior to mouth tube	3
–	Cephalothorax without posterior swelling on ventral surface	5
3	Swimming legs apparently absent	*Peniculus scomberi* Gnanamuthu
–	Swimming legs with 4 pairs	4
4	Trunk about 11 times longer than wide	*Peniculus trichuri* Gnanamuthu
–	Trunk about 8 times longer than wide	*Peniculus stromatei* Gnanamuthu
5	Legs 3 and 4 closer together than legs 1 and 2	*Peniculus communis* Leigh-Sharpe
–	Legs 3 and 4 further apart than legs 1 and 2	6
6	Trunk conical-shaped	*Peniculus furcatus* Krøyer
–	Trunk between 3 and 4.5 times longer than wide	7
7	Mouth tube forming a massive posteriorly-directed proboscis	*Peniculus clavatus* Krøyer
–	Mouth tube not forming a massive posteriorly-directed proboscis	8
8	Cephalothorax ovoid	9
–	Cephalothorax cylindrical	*Peniculus theraponi* Gnanamuthu
–	Cephalothorax widest near posterior margin and tapering anteriorly	*Peniculus elegans* Leigh-Sharpe
9	Abdomen well developed; trunk longer than wide	*Peniculus minuticaudae* Shiino
–	Abdomen well developed; trunk longer than wide; with swelling on the head	10
–	Abdomen reduced; posterior margin of trunk more or less straight	11
10	High degree of ventral swelling on the head; neck constricted	*Peniculus fistula* Nordmann
–	Low degree of ventral swelling on the head; neck constricted	*Peniculus elongatus* Boxshall
11	Trunk 4.3 times longer than wide; neck less than half as long as cephalothorax	*Peniculus ostraciontis* Yamaguti
–	Trunk 3.3 times longer than wide; neck more than half as long as cephalothorax	*Peniculus truncatus* Shiino

### Conflict of interest statement

All authors declare that they do not have any conflict of interest.

## Supplementary Material

XML Treatment for Peniculus minuticaudae

XML Treatment for Peniculus truncatus
